# Interaction between a Sulfated Polysaccharide from Sea Cucumber and Gut Microbiota Influences the Fat Metabolism in Rats

**DOI:** 10.3390/foods12244476

**Published:** 2023-12-14

**Authors:** Yujiao Zhang, Haoran Song, Zhengqi Liu, Chunqing Ai, Chunhong Yan, Xiuping Dong, Shuang Song

**Affiliations:** Liaoning Key Laboratory of Food Nutrition and Health, Collaborative Innovation Center of Seafood Deep Processing, National Engineering Research Center of Seafood, School of Food Science and Technology, Dalian Polytechnic University, Dalian 116034, China; zhangyujiao3@126.com (Y.Z.); 18104099219@163.com (H.S.); liuzhengqi@szu.edu.cn (Z.L.); acqdongying@163.com (C.A.); puniyan@foxmail.com (C.Y.); dxiuping@163.com (X.D.)

**Keywords:** marine polysaccharides, intestinal flora, nontargeted metabolomics

## Abstract

Due to its significant physiological effects, a sulfated polysaccharide has been considered an important nutrient of sea cucumber, but its metabolism in vivo is still unclear. The present study investigated the metabolism of a sea cucumber sulfated polysaccharide (SCSP) in rats and its influence on the metabolite profiles. The quantification by HPLC-MS/MS revealed that the blood level of SCSP achieved a maximum of 54.0 ± 4.8 μg/mL at 2 h after gavage, almost no SCSP was excreted through urine, and 55.4 ± 29.8% of SCSP was eliminated through feces within 24 h. These results prove the utilization of SCSP by gut microbiota, and a further microbiota sequencing analysis indicated that the SCSP utilization in the gut was positively correlated with Muribaculaceae and Clostridia_UCG-014. In addition, the non-targeted metabolomic analysis demonstrated the significant effects of SCSP administration on the metabolite profiles of blood, urine, and feces. It is worth noting that the SCSP supplement decreased palmitic acid, stearic acid, and oleic acid in blood and urine while increasing stearic acid, linoleic acid, and γ-linolenic acid in feces, suggesting the inhibition of fat absorption and the enhancement of fat excretion by SCSP, respectively. The present study shed light on the metabolism in vivo and the influence on the fat metabolism of SCSP.

## 1. Introduction

Sea cucumber is valuable seafood, and it has been considered as a functional food and consumed traditionally in Asian countries, especially in China, Japan, and Korea [1]. A sea cucumber sulfated polysaccharide (SCSP) is the critical nutrient in sea cucumber, and it consists of two sulphated polysaccharides, namely fucosylated chondroitin sulphate and fucoidan sulphate [1,2]. The fucosylated chondroitin sulphate has a chondroitin core and a unique sulfated fucose side chain [3]. Fucoidan sulphate from sea cucumber is a linear polysaccharide consisting of α1→3 linked fucose repeating units with various sulfation patterns [3]. SCSP has significant physiological effects such as anticancer [4], anti-bacterial activity [5], hypolipidemic [6], immune regulation [3,7] and anti-inflammation [8]. However, the underlying mechanism of its effects in vivo is still unclear. It has been proved that SCSP cannot be digested by gastrointestinal enzymes or acid liquid [9,10]. Moreover, as a macromolecule, it is difficult for SCSP to travel through intestinal epithelial cells, which could be inferred through the studies on other macromolecules [11]. Thus, more efforts are needed to reveal the action pathway of SCSP after oral supplementation.

Recently, more and more reports have verified that oral administration of indigestible polysaccharides could regulate gut microbiota so as to benefit the host health [12]. Our previous report has revealed that SCSP can effectively prevent diet-induced obesity through modulating the composition of gut microbiota [13]. It has been proposed that indigestible polysaccharides can be metabolized by gut microbiota to produce bioactive metabolites, such as short-chain fatty acids, demonstrating beneficial effects on host health [14]. However, little evidence for the polysaccharide consumption by gut microbiota in vivo has been reported. Sulfated polysaccharides are more resistant to gut microbiota due to their sulfate groups [15,16]. Our previous study found that a part of SCSP could be fermented by fecal microbiota in vitro. But the utilization degree of SCSP by gut microbiota in vivo is still unknown.

Metabolomics have been widely used to analyze what occurs in diseases’ process by identifying potential biomarkers and the related metabolic pathway [17]. However, increasing evidence has suggested that some metabolites produced by gut microbiota have potent functions for host energy metabolism [18,19]. Non-targeted metabolomics are a comprehensive approach for detecting metabolites as much as possible, and it is more robust in characterizing the metabolism profile and finding involved metabolites without targets [20]. Thus, in the present study, nontargeted metabolomics have been applied to monitor the regulation of SCSP on the metabolites in blood, urine, and feces to reveal the underlying action mechanism of SCSP.

The present study aimed to demonstrate the absorption, metabolism, and excretion of sea cucumber polysaccharides in vivo, and to analyze the effects of SCSP administration on the metabolite profiles of blood, urine, and feces, thus revealing the action pathway of sea cucumber polysaccharides in vivo.

## 2. Materials and Methods

### 2.1. Materials

The sea cucumber sulfated polysaccharide (SCSP) was extracted from *Stichopus japonicus* as previously reported [2]. This fraction (SCSP) was characterized in our previous study. It was composed of fucosylated chondroitin sulfate (179.4 kDa) and fucoidan (>670 kDa) with the mass ratio of 1.00:1.07, as evaluated by gel permeation chromatography (GPC). Furthermore, HPLC analyzed after acid hydrolysis and derivatization with PMP demonstrated that the molar ratio of sulfate/uronic acid/fucose (Fuc)/galactosamine (GalN) in SCSP was 7.6:0.8:9.1:1.0. 1-Phenyl-3-methyl-5-pyrazoline (PMP) was purchased from China National Pharmaceutical Group Co., Ltd. (Beijing, China). Trifluoroacetic acid, ammonium acetate, and formic acid were brought from Aladdin reagent Co., Ltd. (Shanghai, China). 1-Methylnicotinamide-*d*_3_ iodide, acetyl-L-carnitine-(N-methyl-*d*_3_), and DL-glutamic acid were purchased from Cambridge Isotope Laboratories Inc. (Cambridge, CA, USA). 12-[(Cyclohexylcarbamoyl) amino] dodecanoic acid and chondroitin sulfate (shark) were both from Sigma-Aldrich Co. (St. Louis, MI, USA). 

### 2.2. Animals

All animal experiments were performed in accordance with the guidelines for care and use of laboratory animals of Dalian Polytechnic University, and the experiment was approved by the Animal Ethics Committee of Dalian Polytechnic University (ID DLPU2018008).

Eighteen male Sprague Dawley (SD) rats (300 ± 20 g) were purchased from Liaoning Changsheng Biotechnology Company (Benxi, China). The rats were kept under standard conditions with a 12 h light–dark cycle, 23 ± 2 °C ambient temperature, and 55 ± 10% relative humidity. The feed was composed of corn, soybean meal, flour, bran, fish flour, salt, calcium hydrogen phosphate, rock flour, multivitamins, multiple trace elements, amino acids, etc., and more details are shown in Supplemental Table S5. The rats were acclimatized in clean cages for a fortnight prior to the experiment and weighed (310 ± 20 g), and then randomly divided into two groups: the Control group (*n* = 9) and the SCSP group (*n* = 9, named S1~S9).

To prevent the effects of food on SCSP absorption, the rats were fasted for 12 h before SCSP administration but drank water freely. The rats in the SCSP group were gavaged with 150 mg/kg of the SCSP solution (2 mL), and the Control group were gavaged with the same volume of water. Then, the rats were put into metabolic cages and fed normally after 4 h. Fecal and urine samples were collected at 24 h and 48 h (frozen at −80 °C) to ensure that SCSP was wholly excreted. After 1 day, 150 mg/kg of the SCSP solution was gavaged again. Then, the blood was extracted from the tail of the rats at 0 h, 0.5 h, 1 h, 2 h, 4 h, 6 h, 8 h, and 12 h after administration, and placed in centrifuge tubes containing an EDTA anticoagulant. At 24 h, the rats were anesthetized with ether, and blood samples were collected from the orbital vein and placed in centrifuge tubes containing the EDTA anticoagulant and ordinary centrifuge tubes. The coagulation time of the blood collected at 2 h after gavage from the broken tail of rats using a capillary was measured according to previously described reports [21,22].

### 2.3. Quantification of SCSP in Plasma, Feces, and Urine

Firstly, SCSP in plasma and feces was isolated. Plasma samples (0.1 mL) were suspended in a mixture of 0.5 mL of 2.6 M trifluoroacetic acid (TFA) and 0.4 mL of water. Dried fecal samples (0.20 g) were dissolved in 4 mL of water, and after centrifugation at 8000 rpm for 10 min, the supernatant (1 mL) was collected and mixed with ethanol (4 mL) and kept at 4 °C overnight. Then, the precipitate was collected after centrifugation (4000 rpm, 10 min). 

Quantification of SCSP was conducted as previously described [2]. Briefly, the collected precipitate containing SCSP was redissolved in water (5 mL). After that, 0.5 mL of the solution was mixed with 0.5 mL of 2.6 M TFA. Urine samples (10 mL) were concentrated to 1 mL using a rotary evaporator at 50 °C under a vacuum. Then, a four-fold volume of ethanol was added, and the mixture stood at 4 °C overnight. After that, the precipitate was collected by centrifugation (4000 rpm, 10 min), and dissolved in 1 mL of 1.3 M TFA. 

Immediately after the above treatment, the samples were heated at 105 °C for 3 h in sealed tubes for the acid hydrolysis. Then, the resulting solutions were dried with a vacuum concentrator (LaboGene Aps., Lynge, Denmark). Then, 0.5 mL of methanol was added to the solution, which was dried again. This procedure was repeated thrice to remove TFA thoroughly. Then, 100 μL of 1 mg/mL lactose was added as an internal standard, and the mixtures were resolved in a solution composed of 400 μL of ammonia and 400 μL of a 0.3 M PMP methanolic solution. After heating at 70 °C for 30 min in a water bath, the solution was dried with the vacuum concentrator. Thus, the PMP derivatives of hydrolysates of SCSP were prepared for an ESI-MS/MS analysis. 

The chromatography separation was carried out on a Shimadzu HPLC system (LC-20ADXR pumps, SIL-20AXP Autosampler, Kyoto, Japan) with a Thermo Scientific (Waltham, MA, USA) Hypersil Gold column (150 × 2.1 mm, 5 μm). A 20 mM ammonium acetate solution and acetonitrile (85:15, *v*/*v*) were used as the mobile phase at a flow rate of 0.5 mL/min, and the column temperature was maintained at 30 °C. The ESI-MS/MS analysis was performed on a Q-trap 4000 triple-quadrupole mass spectrometer (AB Sciex, Framingham, MA, USA) in the positive-ion multiple reaction monitoring (MRM) mode. Mass spectrometry analysis parameters are provided in Supplemental Table S4.

### 2.4. Sequencing Analysis of the Gut Microbiota

Sequencing was carried out on an Illumina MiSeq PE250 platform (Novogene Genomics Technology Co., Ltd., Beijing, China, https://magic.novogene.com (accessed on 1 June 2021)). Briefly, Genomic DNA was isolated from fecal samples by the sodium dodecyl sulfate (SDS) method and assayed using agarose gel electrophoresis. After that, the genomic DNA was amplified with the 341F (5′-CCTACGGGNGGCWGCAG-3′) and 806R (5′-GGACTACHVGGGTWTCTAAT-3′) primers specific for the V3–V4 region of the 16S rRNA gene. Sequencing libraries were generated using the Ion Plus Fragment Library Kit 48 rxns and assessed on the Qubit@ 2.0 fluorometer (Thermo Scientific, Waltham, MA, USA). Alpha diversity and rarefaction curves were analyzed using QIIME V1.7.0. Venn diagrams were constructed using R software package V3.0.3 and were used to compare and analyze differences in the composition of OTUs in the intestinal flora between groups. A principal coordinate analysis of PCoA and β-diversity was calculated using QIIME software V1.7.0 and presented using Stat, Ggplot2, and WGCNA packages in R software V2.15.3.

### 2.5. Analysis of Metabolites by UPLC-Q-TOF MS

According to the reported protocols [23], the serum sample (50 μL), the urine sample (150 μL), and the fecal sample (300 mg) were mixed with 200 μL of methanol (−20 °C), 300 μL of water, and 500 μL of a mixture (acetonitrile/methanol/water, 2:2:1, *v*/*v*/*v*), respectively. Then, 10 μL of a mixed internal standard solution containing 20 ng/mL of 12-[(cyclohexylcarbamoyl) amino] dodecanoic acid, 210 ng/mL of 1-methylnicotinamide-*d*_3_ iodide, 51 ng/mL of acetyl-L-carnitine-(N-methyl-*d*_3_), and 3500 ng/mL of DL-glutamic acid (2,4,4-*d*_3_, 98%) was added. The mixture was vortexed for 30 s, and then stored in a refrigerator at −20 °C for 30 min. After that, the mixture was centrifuged at 12,000 g/min for 5 min at 4 °C and the supernatant was collected for a UPLC-Q-TOF MS analysis.

UPLC-Q-TOF MS analyses were performed on AB Sciex Triple TOF 5600 using both positive ion (PI) and negative ion (NI) modes. The chromatography separation was carried out using Waters Xselect @HSS T3 (2.5 μm, 100 mm × 2.1 mm) at a flow rate of 0.4 mL/min, and the column temperature was maintained at 30 °C. For the PI mode, the mobile phase was eluent A (0.1% formic acid in water, *v*/*v*) and eluent B (acetonitrile with 0.1% formic acid, *v*/*v*). For the NI mode, the mobile phase was eluent A (5 mM ammonium acetate in water) and eluent B (5 mM ammonium acetate in water/acetonitrile = 10:90, *v*/*v*). The solvent gradient was set as follows: 0–5 min, 0–20% B; 5–7 min, 20% B; 7–14 min, 20–100% B. The quality Control samples (QC) were prepared by mixing equal (10 μL) amounts of each serum sample. The AB Sciex triple TOF 5600 mass spectrometer was operated with ion source gas 1 of 55 arb, ion source gas 2 of 55 arb, curtain gas of 35 arb, a temperature of 550 °C, and ion spray voltage floating of 5500 V for the PI mode and −4500 V for the NI mode.

The collected raw data were converted to “abf” format and the converted files were imported into MSDIAL. A principal component analysis (PCA) and orthogonal partial least squares discriminant analysis (OPLS-DA) were performed with SIMCA 14.1 software after exportation. And then the database in MSDIAL was used to identify the metabolites with a VIP value greater than 1.0.

### 2.6. Statistical Analysis

All data are expressed as means ± standard deviations (SDs). Student’s *t*-test was used to evaluate the difference between two groups, and three groups were made by one-way ANOVA with Duncan’s test. *p* < 0.05 was considered to be significantly different. A multiple linear regression analysis was used to investigate the relationship between the utilization of SCSP and bacterial OTUs. Bacterial OUTs with >1% abundance were included in the regression model.

## 3. Results and Discussion

### 3.1. Absorption and Exertion of SCSP in Rats

**Figure 1:**
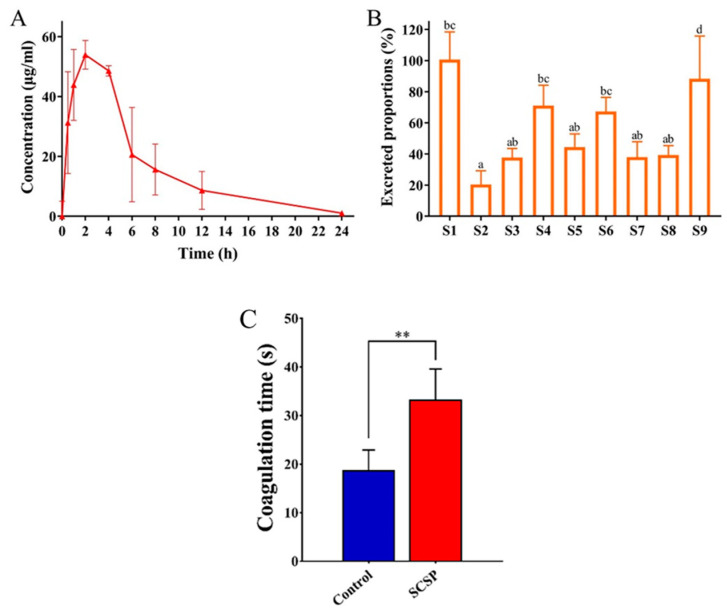
Mean plasma concentration–time profile of SCSP in rats after the gavage (*n* = 3, (**A**)), the excreted proportions of SCSP through feces within 24 h in different rat individuals (**B**), and the effect of SCSP on coagulation time in rats (*n* = 3, ** *p* < 0.01, (**C**)). S1 to S9 stand for 9 rats making up different individuals. Graph bars marked with different letters on top represent statistically significant results (*p* < 0.05) based on one-way analysis of variance (ANOVA) followed by Duncan’s test, whereas bars labelled with the same letter correspond to results that show no statistically significant differences.

In order to reveal the metabolism of SCSP in rats, SCSP in plasma, feces, and urine of rats after the gavage (46.5 mg) was quantified by HPLC-MS/MS. The method for the quantification of SCSP was validated for sensitivity, recovery, and repeatability (Supplemental Results). The results showed that the blood concentration of SCSP reached the maximum (54.0 ± 4.8 μg/mL) at 2 h (Figure 1A). Since the circulating blood volume of rats is generally about 11 mL, it could be estimated that 1.2% SCSP was in blood circulation at that time. As shown in Table 1, SCSP in urine samples collected within 0~24 h and 24~48 h was nearly undetectable, and no more than 0.2% SCSP was excreted through urine as polymers. The same phenomenon has been previously reported [24]. Of note, 55.4 ± 29.8% of SCSP was detected in fecal samples within 24 h although there was great variability among individuals (Figure 1B). However, SCSP was barely detectable in feces after 24 h. From the above results, it could be concluded that the SCSP was mainly eliminated via feces within 24 h. Interestingly, a considerable proportion (~44%) of SCSP disappeared, indicating its degradation by intestinal flora. It has been reported that SCSP could be partially degraded by intestinal flora [25,26,27]. Moreover, the differences of SCSP contents in feces among rat individuals could be contributed to their diversity of intestinal microbiota communities [28]. As shown in Figure 1C, SCSP significantly prolonged coagulation time compared to the Control group (*p* < 0.01). Nadezhda E. Ustyuzhanina and R.J.C. Fonseca et al. had proved that fucosylated chondroitin sulfate in sea cucumber had an obvious anticoagulant effect by in vitro and in vivo experiments [29,30]. Our experiments also demonstrated the anticoagulant effect of gavaged SCSP, indicating that SCSP could be absorbed into the systemic circulation.

### 3.2. Relationship between Intestinal Flora and SCSP Utilization

**Figure 2:**
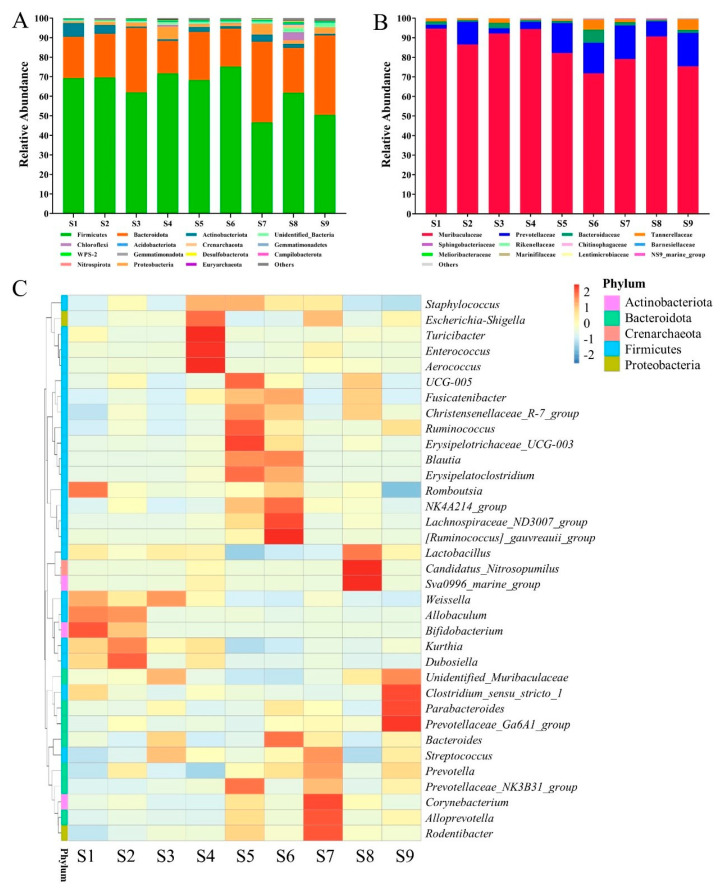
The relative abundances of intestinal microflora in different rats at the phylum level (**A**), the relative abundances of different families in Bacteroidota (**B**), and the heatmap of top 35 genera (**C**).

The gut microbiota of the rats were analyzed through multiplex sequencing covering the V3–V4 regions of 16S rRNA, and the differences in the microbiota of the rat individuals were demonstrated. The relative abundances of microbiota at the phylum level (Figure 2A) showed that Bacteroidetes and Firmicutes were the dominant intestinal bacteria in these rats. The composition of Bacteroidetes was further demonstrated as shown in Figure 2B. Muribaculaceae was the most abundant family in Bacteroidetes, with an abundance of 85.2%, followed by Prevotellaceae (~10%) and Bacteroideae (~1%). Furthermore, the relative contributions of 35 dominant genera showed in the heatmap (Figure 2C) revealed that the composition of the intestinal flora varied greatly among individuals. The correlation between Operational Taxonomic Units (OTUs) and the utilization rate of SCSP was demonstrated by a multiple linear regression analysis (Supplemental Table S3) to obtain the regression model y = 35.164x_1_ + 38.257x_2_ − 0.145, where x_1_ and x_2_ are from Muribaculaceae and Clostridia_UCG-014, respectively. Thus, the result indicates the two OTUs were both positively correlated with the utilization rate of SCSP (*p* < 0.05).

SCSP metabolism is a complex process that requires the participation of many gut microbes. It has been reported that some microorganisms such as *Bacteroides* and *Parabacteroides* are also involved in the metabolism of SCSP [27]. Different gut microbiota structures could respond to the intake food differently [27,31]. The discrepancy in the SCSP utilization in these rats could be attributed to the great diversity of their microbiota communities. Bacteroidetes, including Muribaculaceae, possess very large numbers of genes encoding carbohydrate-active enzymes, and are considered as the main contributors for polysaccharide utilization [32,33]. Muribaculaceae widely exist in the gut microbiota and a genome analysis indicated their potential capability to degrade complex carbohydrates [34]. Clostridia_UCG-014 has been identified as a member of Clostridiaceae, which possessed some well-studied cellulolytic organisms [35], and it has been considered as one of the major microbial contributors to digest polysaccharides due to its carbohydrate-degrading genes [36]. Then, the present study will suggest some bacteria from Muribaculaceae and Clostridiaceae that are keystone bacteria for SCSP utilization in the gut, affecting SCSP’s outcome, such as anti-obesity and anti-type 2 diabetes [37].

### 3.3. Regulation of SCSP on Metabolite Profile of Feces

**Figure 3:**
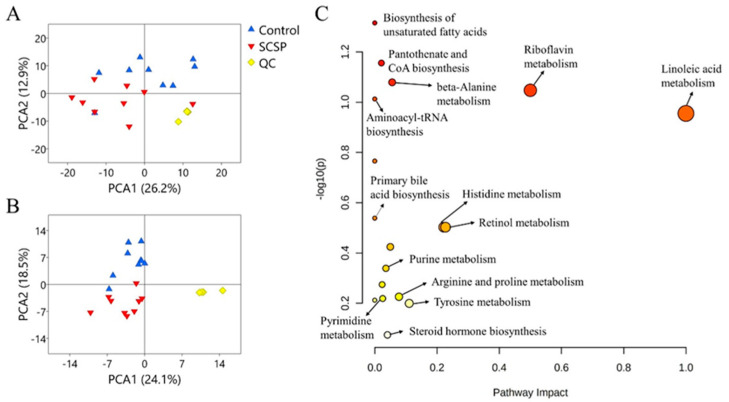
PCA analysis of the feces metabolites detected in the positive (**A**) and negative (**B**) modes and metabolism pathway analysis in rat feces (**C**).

The effect of SCSP administration on the fecal metabolites was investigated through a non-targeted metabolomic analysis using Q-TOF-MS. As shown by PCA (Figure 3A) and OPLS-DA (Supplemental Figure S3), the fecal metabolite profiles of the SCSP group and the Control group separated with each other, indicating SCSP changed the metabolic profiles in the host gut. In total, 95 differential metabolites were identified in feces (Table 2 and Table 3), and the related metabolic pathways with -log10 (p) and impact as the horizontal and vertical coordinates are shown in Figure 3B. The metabolic pathway obviously influenced by the SCSP supplement includes biosynthesis of unsaturated fatty acids, pantothenate and CoA biosynthesis, β-alanine metabolism, aminoacyl-tRNA biosynthesis, primary bile acid biosynthesis, riboflavin metabolism, linoleic acid metabolism, purine metabolism, histidine metabolism, retinol metabolism, arginine and proline metabolism, pyrimidine metabolism, tyrosine metabolism, and steroid hormone biosynthesis. Interestingly, the biosynthesis of unsaturated fatty acids and linoleic acid metabolism were both related to lipid metabolism. Of note, stearic acid, linoleic acid, and γ-linolenic acid in these two pathways were enriched by SCSP, indicating the enhancement of lipid metabolism in the guts of rats administrated SCSP. 

As we know, diet fat is digested and absorbed in the stomach and the small intestine [38]. More active lipid metabolism in the guts suggests more fat was transported to the gut without being absorbed by the upper digestive tract. Thus, the results in the present study indicate the supplementation of SCSP could inhibit the fat absorption. It has been reported that SCSP could prevent obesity induced by a high-fat diet [13], and the inhibition effect of SCSP against the fat absorption is one of the possible reasons for its anti-obesity activity. Liu et al. reported that the gut microbiota in HFD-fed mice were dominated by Muribaculaceae species, and the untargeted metabolomics analyses of fecal samples show that microbial changes altered the bacteria-derived metabolites, which related to linoleic acid metabolism [39]. Thus, it can be inferred that SCSP could interfere with linoleic acid metabolism by affecting Muribaculaceae to exert anti-obesity effects.

### 3.4. Regulation of SCSP on Metabolite Profiles of Serum and Urine

**Figure 4:**
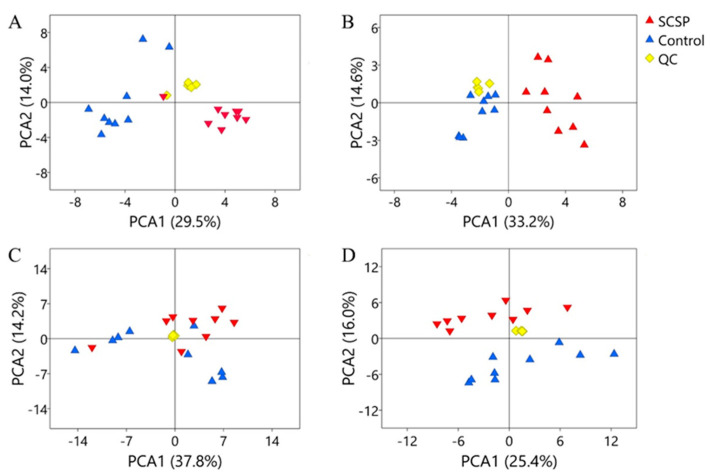
PCA analysis of the serum and urine metabolites detected in the positive (**A**,**C**) and negative (**B**,**D**) modes.

To evaluate the physiological effect of single gavage of SCSP, the metabolites in serum and urine of the rats were also determined by UPLC-Q-TOF MS in both positive and negative modes. As shown in Table 4, Table 5, Table 6 and Table 7, 17 and 39 differential metabolites were identified in the serum and urine, respectively. The PCA analysis demonstrated a distinction between the serum metabolite profiles of the Control and SCSP groups in both of the positive (Figure 4A) and negative modes (Figure 4B). Moreover, although no significant separation was observed in urine profiles of the Control and SCSP groups detected in the positive mode (Figure 4C), the analysis result determined in the negative mode that an obvious separation was exhibited (Figure 4D). The separation between Control and SCSP groups demonstrated in the negative mode could be attributed to some acidic compounds, such as glycolic acid and arachidic acid. The metabolism pathway analysis in serum and urine metabolites is shown in Supplemental Figure S4. Thus, it can be concluded that single-dose SCSP administration can affect the in vivo metabolism of rats.

Of note, palmitic acid, stearic acid, and oleic acid, which were all increased in the feces of the SCSP group, all decreased in serum and urine of the SCSP group. These long-chain fatty acids (LCFAs) are components of pork fat in the fodder, and they could be absorbed into serum [40]. Thus, the less serum LCFAs in the SCSP group indicated that SCSP could inhibit the LCFAs’ absorption so as to increase fat excretion via feces. It has been reported that the dietary supplementation of SCSP could significantly reduce fat accumulation and lipid levels in high-fat-diet-fed mice [41]. Then, the findings in the present study suggest the inhibition of LCFAs’ absorption plays a role in the anti-obesity and hypolipidemic effects of SCSP. It has been well documented that dietary fibers could prevent the absorption of fat by chelation with their micro-molecular chains [42]. Then, it could be inferred these SCSP micromolecules may bind to fat in the intestine so as to interfere with the fat metabolism in the host.

Taking together all the above results, single gavage of SCSP in vivo could be proposed as follows: a proportion of SCSP could participate in various metabolic reactions of the rats to produce different metabolites, thus benefiting the host health. The other SCSP was not degraded, but they could reduce obesity and hyperlipidemia by inhibiting fat absorption and increasing fat excretion into the feces. This is consistent with the previous research result that SCSP can prevent diet-induced obesity and its associated diseases [13].

## 4. Conclusions

The present study demonstrated the absorption, the exertion, and the metabolism by gut microbiota of SCSP. As shown by the quantitative analysis of SCSP in blood, urine, and feces, only a small amount of SCSP could be absorbed into the blood while a proportion of SCSP was excreted in the feces, which suggests that a considerable proportion of SCSP was metabolized by gut microbiota and the metabolized amount of SCSP varied among individuals. The SCSP degradation in the intestine was positively correlated with Muribaculaceae and Clostridia_UCG-014, confirming the involvement of these gut bacteria in metabolism of SCSP in the gut. Furthermore, the changes of the metabolites in blood, urine, and feces caused by SCSP administration revealed that SCSP could inhibit the fat absorption by increasing fat excretion. Our findings provide a better understanding of the action mechanism of SCSP in vivo.

## Figures and Tables

**Table 1:** Excreted amounts and ratios of SCSP in feces and urine.

Sample	Collecting Time (h)	0~24	24~48
Feces (*n* = 9)	SCSP amount (mg)	27.267 ± 14.662	0.0604 ± 0.083 ^a^
	Excretion ratio (%)	55.42 ± 29.80	0.15 ± 0.17
Urine (*n* = 3)	SCSP amount (mg)	<0.1 ^a^	<0.1 ^a^
	Excretion ratio (%)	<0.2	<0.2

^a^ The data were not significantly different from those in Control group.

**Table 2:** Identification results of differential metabolites in feces detected in the positive ion mode.

No.	Name	Formula	VIP	SCSP vs. Control
1	3-Amino-1,2,4-triazole	C_2_H_4_N_4_	1.37074	↑ (***)
2	Morpholine	C_4_H_9_NO	6.33961	↓
3	Diethanolamine	C_4_H_11_NO_2_	1.32672	↓ (**)
4	Dihydrouracil	C_4_H_6_N_2_O_2_	1.14054	↑ (*)
5	L-proline	C_5_H_9_NO_2_	2.47750	↑ (***)
6	Valine	C_5_H_11_NO_2_	1.17142	↑ (*)
7	Cinnamaldehyde	C_9_H_8_O	1.89465	↓ (**)
8	2-Aminobenzimidazole	C_7_H_7_N_3_	1.41216	↑ (***)
9	Hypoxanthine	C_5_H_4_N_4_O	1.58532	↓ (**)
10	Guanine	C_5_H_5_N_5_O	1.22418	↓ (**)
11	His	C_6_H_9_N_3_O_2_	1.14781	↑ (**)
12	2,8-Quinolinediol	C_9_H_7_NO_2_	2.45795	↑ (**)
13	Metronidazole	C_6_H_9_N_3_O_3_	3.82603	↑ (*)
14	7,8-Dihydroxycoumarin	C_9_H_6_O_4_	1.67340	↑ (*)
15	5-Butylpyridine-2-carboxylic acid	C_10_H_13_NO_2_	1.14137	↓
16	Metamitron-desamino	C_10_H_9_N_3_O	1.49271	↓
17	Levodopa	C_9_H_11_NO_4_	1.56546	↑
18	2-(4-Isobutylphenyl) propionic acid	C_13_H_18_O_2_	1.45771	↓ (*)
19	Pilocarpine	C_11_H_16_N_2_O_2_	2.66077	↓ (***)
20	Terbumeton	C_10_H_19_N_5_O	1.10183	↓ (*)
21	Flonicamid	C_9_H_6_F_3_N_3_O	3.03765	↓ (**)
22	Lumichrome	C_12_H_10_N_4_O_2_	2.85914	↑ (**)
23	(2Z)-2-Benzylidene-6-methoxy-1-benzofuran-3(2H)-one	C_16_H_12_O_3_	1.40162	↓ (*)
24	Dehydroepiandrosterone	C_19_H_28_O_2_	2.13486	↓
25	Daidzein	C_15_H_10_O_4_	1.41342	↑ (**)
26	Epiandrosterone	C_19_H_30_O_2_	1.03079	↑
27	Jaeschkeanadiol	C_15_H_26_O_2_	1.06159	↓
28	Huperzine A	C_15_H_18_N_2_O	2.14980	↑
29	Graveolide	C_15_H_20_O_3_	1.38494	↑ (**)
30	Baicalein	C_15_H_10_O_5_	1.68694	↓ (**)
31	Galaxolidone	C_18_H_24_O_2_	1.03394	↑ (*)
32	(-)-Eburnamonine	C_19_H_22_N_2_O	1.93955	↑ (***)
33	All-trans-retinoic acid	C_20_H_28_O_2_	1.40410	↑ (*)
34	Methyl 3-(3,4-dihydroxy-5-phenyloxolan-2-yl)-3-hydroxypropanoate	C_14_H_18_O_6_	1.82178	↑
35	5-(4-Hydroxybenzyl)-4-(2-hydroxy-4-methoxyphenyl)-2(5H)-furanone	C_18_H_16_O_5_	1.05768	↓ (**)
36	5-(1,2,4a,5-Tetramethyl-7-oxo-3,4,8,8a-tetrahydro-2H-naphthalen-1-yl)-3-methylpentanoic acid	C_20_H_32_O_3_	1.23648	↓ (***)
37	Hydroquinidine	C_20_H_26_N_2_O_2_	1.19523	↑ (***)
38	Raclopride	C_15_H_20_Cl_2_N_2_O_3_	1.19604	↓ (*)
39	Eicosanoids–bicycloPGE1	C_20_H_32_O_4_	2.81380	↓ (***)
40	Aphidicolin	C_20_H_34_O_4_	1.36684	↓ (*)
41	Melibiose	C_12_H_22_O_11_	2.25455	↓
42	(2E,4E)-12-Hydroxy-13-(hydroxymethyl)-3,5,7-trimethyltetradeca-2,4-dienedioic acid	C_18_H_30_O_6_	1.02162	↓
43	Hirsutine	C_22_H_28_N_2_O_3_	1.12443	↓ (**)
44	4-(Hydroxymethyl)-1-isopropyl-3-cyclohexen-1-yl beta-d-glucopyranoside	C_16_H_28_O_7_	1.00738	↓
45	3-Methyl-5-(5,5,8a-trimethyl-2-methylene-7-oxodecahydro-1-naphthalenyl) pentyl acetate	C_22_H_36_O_3_	1.69849	↓
46	Deoxycholic acid	C_24_H_40_O_4_	1.00172	↑ (**)
47	(-)-Riboflavin	C_17_H_20_N_4_O_6_	1.38226	↓ (*)
48	Methyl robustone	C_22_H_18_O_6_	3.32767	↑ (*)
49	Lagochilin	C_20_H_36_O_5_	1.12443	↓ (*)
50	Colladonine	C_26_H_32_O_5_	1.28344	↓ (**)
51	Voacristine	C_22_H_28_N_2_O_4_	1.24153	↓ (**)
52	Cholic acid	C_24_H_40_O_5_	1.03719	↓
53	Monolinolein	C_21_H_38_O_4_	1.69026	↑ (**)
54	11,12-Methylenedioxykopsinaline	C_22_H_26_N_2_O_5_	1.80736	↓
55	6-Hydroxy-2,4,4-trimethyl-3-(3-oxobutyl)-2-cyclohexen-1-yl beta-d-glucopyranoside	C_19_H_32_O_8_	1.74942	↓ (***)
56	2-Acetoxy-4-pentadecylbenzoic acid	C_24_H_38_O_4_	1.32797	↓
57	4,6′,7′-Trihydroxy-6-(hydroxymethyl)-2′,5′,5′,8a′-tetramethyl-3′,4′,4a′,5′,6′,7′,8′,8a′-octahydro-2′H,3H-spiro[1-benzofuran-2,1′-naphthalene]-7-carbaldehyde	C_23_H_32_O_6_	4.20631	↑
58	Lovastatin M + Na	C_24_H_36_O_5_	1.20112	↓
59	Irbesartan	C_25_H_28_N_6_O	1.00854	↓
60	Hyocholic acid	C_24_H_40_O_5_	2.79818	↓
61	Beta-peltatin	C_22_H_22_O_8_	3.54980	↑
62	3-[5-Hydroxy-7-methoxy-2,3-dimethyl-6-(3-methylbut-2-enyl)-4-oxo-2,3-dihydrochromen-8-yl] hexanoic acid	C_23_H_32_O_6_	4.08549	↑ (**)
63	5-Hydroxy-5-(2-hydroxy-2-propanyl)-3,8-dimethyl-2-oxo-1,2,4,5,6,7,8,8a-octahydro-6-azulenyl β-d-glucopyranoside	C_21_H_34_O_9_	1.03474	↑
64	6-[3-[(3,4-Dimethoxyphenyl) methyl]-4-methoxy-2-(methoxymethyl) butyl]-4-methoxy-1,3-benzodioxole	C_24_H_32_O_7_	1.05174	↓
65	Prednisolone_tebutate	C_27_H_38_O_6_	1.53337	↓ (*)
66	Lunarine	C_25_H_31_N_3_O_4_	1.10489	↓
67	Lycoctonine	C_25_H_41_NO_7_	1.02154	↓ (*)
68	NCGC00385237-01-C30H48O4	C_30_H_48_O_4_	1.03038	↓ (*)
69	Petunidin-3-O-beta-glucoside	C_22_H_23_O_12_	1.63603	↑ (*)
70	Emetine	C_29_H_40_N_2_O_4_	1.08634	↓
71	(2R)-2-Hydroxy-3-(palmitoyloxy) propyl 2-(trimethylammonio)ethyl phosphate	C_24_H_50_NO_7_P	1.13281	↓ (**)
72	1-Linoleoyl-2-hydroxy-sn-glycero-3-PC	C_26_H_50_NO_7_P	1.45727	↓
73	Plasma ID-2759	C_26_H_52_NO_7_P	1.05955	↓ (**)
74	Rhusflavone	C_30_H_22_O_10_	1.18525	↑
75	(1R,2R,3R,3aS,5aS,6R,7R,10R,10aR,10cR)-1,2,6,7-Tetrahydroxy-10a,10c-tetramethyl-4-oxo-6a,7,10,10a,10b,10c-dodecahydro-1H-phenanthro[10,1-bc] furan-10-yl beta-d-glucopyranoside	C_25_H_38_O_12_	2.23274	↓ (*)
76	Pantethine	C_22_H_42_N_4_O_8_S_2_	1.16095	↓
77	Cyanidin-3-O-sophoroside	C_27_H_31_O_16_	1.42677	↑ (*)
78	L-Glutathione	C_20_H_32_N_6_O_12_S_2_	1.06517	↑ (*)
79	Gitoxin	C_41_H_64_O_14_	3.23925	↑ (**)

Differential metabolites were analyzed using unpaired two-tailed Student’s *t*-test with Bonferroni correction (* *p* < 0.05, ** *p* < 0.01, and *** *p* < 0.001 vs. the Control group; ↑, increase; ↓, decrease).

**Table 3:** Identification results of differential metabolites in feces detected in the negative ion mode.

No.	Name	Formula	VIP	SCSP vs. Control
1	Glyceraldehyde	C_3_H_6_O_3_	1.02494	↑ (**)
2	4-Hydroxybenzaldehyde	C_7_H_6_O_2_	1.48141	↓
3	Cis-Muconic acid	C_6_H_6_O_4_	1.90860	↓ (***)
4	Laurilsulfate	C_12_H_26_O_4_S	1.70544	↓
5	γ-Linolenic acid	C_18_H_30_O_2_	1.68250	↑
6	Linoleic acid	C_18_H_32_O_2_	5.62134	↑ (***)
7	Stearic acid	C_18_H_36_O_2_	1.40114	↑ (*)
8	9,10-DiHOME	C_18_H_34_O_4_	1.48835	↓ (*)
9	7,15-Dihydroxyabieta-8,11,13-trien-18-oic acid	C_20_H_28_O_4_	1.11551	↓ (*)
10	5-[2-(Furan-3-yl) ethyl]-8-hydroxy-5,6,8a-trimethyl-3,4,4a,6,7,8-hexahydronaphthalene-1-carboxylic acid	C_20_H_28_O_4_	1.29029	↓
11	Lithochol-11-enic acid	C_24_H_38_O_3_	2.07634	↑ (**)
12	Lithocholic acid	C_24_H_40_O_3_	2.28879	↑ (**)
13	Deoxycholic acid	C_24_H_40_O_4_	4.56029	↓
14	Chenodiol	C_24_H_40_O_4_	5.97657	↓
15	Hyocholic acid	C_24_H_40_O_5_	6.87300	↓
16	Irbesartan	C_25_H_28_N_6_O	1.39312	↑ (*)

Differential metabolites were analyzed using unpaired two-tailed Student’s *t*-test with Bonferroni correction (* *p* < 0.05, ** *p* < 0.01, and *** *p* < 0.001 vs. the Control group; ↑, increase; ↓, decrease).

**Table 4:** Identification results of differential metabolites in serum detected in the positive ion mode.

No.	Name	Formula	VIP	SCSP vs. Control
1	Glycocyamine	C_3_H_7_N_3_O_2_	1.84134	↓ (*)
2	3-Formylindole	C_9_H_7_NO	3.12390	↑
3	Phosphocholine	C_5_H_15_NO_4_P	2.46685	↓ (***)
4	Phytosphingosine	C_18_H_39_NO_3_	1.55859	↑ (***)
5	Iprovalicarb	C_18_H_28_N_23_	3.53042	↓
6	LysoPC (18:3(9Z,12Z,15Z))	C_26_H_48_NOP	1.57622	↓ (***)
7	1-Linoleoyl-2-hydroxy-sn-glycero-3-PC	C_26_H_50_NOP	1.33664	↑
8	Isohernandezine	C_39_H_44_N_27_	1.41083	↓
9	(4R,9β,23E)-2-(β-d-Glucopyranosyloxy)-16,20-dihydroxy-9,10,14-trimethyl-1,11,22-trioxo-4,9-cyclo-9,10-secocholesta-2,5,23-trien-25-yl acetate	C_38_H_54_O_13_	1.02230	↑ (***)

Differential metabolites were analyzed using unpaired two-tailed Student’s *t*-test with Bonferroni correction (* *p* < 0.05, and *** *p* < 0.001 vs. the Control group; ↑, increase; ↓, decrease).

**Table 5:** Identification results of differential metabolites in serum detected in the negative ion mode.

No.	Name	Formula	VIP	SCSP vs. Control
1	Lactic acid	C_3_H_6_O_3_	1.89469	↑ (*)
2	Palmitic Acid	C_16_H_32_O_2_	2.12223	↑ (**)
3	Oleic acid	C_18_H_34_O_2_	1.05400	↑
4	Stearic acid	C_18_H_36_O_2_	1.23960	↑
5	Thymol-β-d-glucoside	C_16_H_24_O_6_	1.62896	↑
6	9,10-DiHOME	C_18_H_34_O_4_	1.19020	↑ (**)
7	Hydroquinidine	C_20_H_26_N_2_O_2_	1.72053	↑ (***)
8	19S-Methoxytubotaiwine	C_21_H_26_N_2_O_3_	1.52218	↑

Differential metabolites were analyzed using unpaired two-tailed Student’s *t*-test with Bonferroni correction (* *p* < 0.05, ** *p* < 0.01, and *** *p* < 0.001 vs. the Control group; ↑, increase).

**Table 6:** Identification results of differential metabolites in urine detected in the positive ion mode.

No.	Name	Formula	VIP	SCSP vs. Control
1	Pyrrolidine	C_4_H_9_N	1.22976	↑ (***)
2	3-Amino-1,2,4-triazole	C_2_H_4_N_4_	3.94189	↑ (**)
3	D-Alanine	C_3_H_7_NO_2_	1.19995	↑ (*)
4	5-Methylcytosine	C_5_H_7_N_3_O	2.25112	↑ (***)
5	2-Benzoxazolinone	C_7_H_5_NO_2_	1.82869	↑
6	3-Methyladenine	C_6_H_7_N_5_	1.69258	↑ (**)
7	7-Methanesulfinylheptanenitrile	C_8_H_15_NOS	1.57761	↑ (*)
8	Ethyl-4-dimethylaminobenzoate	C_11_H_15_NO_2_	2.53641	↑ (**)
9	Monuron	C_9_H_11_C_l_N_2_O	1.31887	↑ (*)
10	Thiabendazole	C_10_H_7_N_3_S	1.43739	↓
11	Cyclo(proline–leucine)	C_11_H_18_N_2_O_2_	1.25286	↑ (**)
12	Mefenamic acid	C_15_H_15_NO_2_	1.15207	↑
13	Estriol	C_18_H_24_O_3_	1.84057	↑ (**)
14	Daidzein	C_15_H_10_O_4_	1.88659	↓
15	Genistein	C_15_H_10_O_5_	1.82289	↑ (*)
16	Glycitein	C_16_H_12_O_5_	1.03938	↑
17	4′-Methylgenistein	C_16_H_12_O_5_	1.22976	↑ (*)
18	Adenosine 5′-monophosphate	C_10_H_14_N_5_O_7_P	2.49862	↑
19	Methyl (2E,4E,8E)-7,13-dihydroxy-4,8,12-trimethyltetradeca-2,4,8-trienoate	C_18_H_30_O_4_	1.03817	↑ (*)
20	2-(2,6-Dihydroxy-4-methoxycarbonylbenzoyl)-3-hydroxybenzoic acid	C_16_H_12_O_8_	1.27008	↑

Differential metabolites were analyzed using unpaired two-tailed Student’s *t*-test with Bonferroni correction (* *p* < 0.05, ** *p* < 0.01, and *** *p* < 0.001 vs. the Control group; ↑, increase; ↓, decrease).

**Table 7:** Identification results of differential metabolites in urine detected in the negative ion mode.

No.	Name	Formula	VIP	SCSP vs. Control
1	Glycolic acid	C_2_H_4_O_3_	1.41701	↑ (***)
2	Catechol	C_6_H_6_O_2_	2.64152	↓
3	5-Methyl-1H-benzotriazole	C_7_H_7_N_3_	1.32223	↓
4	Ortho-aminobenzoic acid	C_7_H_7_NO_2_	1.17925	↓ (*)
5	4-Hydroxyquinoline	C_9_H_7_NO	1.07799	↓
6	2-Hydroxyacetanilide	C_8_H_9_NO_2_	1.25584	↑ (*)
7	Divarinol	C_9_H_12_O_2_	1.62192	↑ (***)
8	Allantoin	C_4_H_6_N_4_O_3_	1.14447	↓
9	Saccharin	C_7_H_5_NO_3_S	1.13550	↑
10	4-Pyridoxic acid	C_8_H_9_NO_4_	2.62819	↓
11	3-Indoxyl sulfate	C_8_H_7_NO_4_S	1.82190	↓
12	Pantothenate	C_9_H_17_NO_5_	1.01748	↑ (*)
13	Daidzein	C_15_H_10_O_4_	2.82778	↑ (***)
14	9-Trans-palmitelaidic acid	C_16_H_30_O_2_	1.01288	↓ (***)
15	Trans-vaccenic acid	C_18_H_34_O_2_	4.77431	↓ (***)
16	Oleic acid	C_18_H_34_O_2_	3.37721	↓ (***)
17	Stearic acid	C_18_H_36_O_2_	2.22722	↓ (***)
18	8-(3-Octyl-2-oxiranyl) octanoic acid	C_18_H_34_O_3_	1.59172	↓ (***)
19	Arachidic acid	C_20_H_40_O_2_	1.36823	↓ (***)

Differential metabolites were analyzed using unpaired two-tailed Student’s *t*-test with Bonferroni correction (* *p* < 0.05, and *** *p* < 0.001 vs. the Control group; ↑, increase; ↓, decrease).

## Data Availability

Data is contained within the article or supplementary material.

## Supplementary Materials

The following supporting information can be downloaded at: https://www.mdpi.com/article/10.3390/foods12244476/s1. Supplemental Results 1 The validation of the method for quantification of SCSP. Supplemental Table S1 The LOD and LOQ of solid samples (dried sea cucumber) and liquid samples (sea cucumber wine). Supplemental Table S2 The recoveries of SCSP in solid samples (dried sea cucumber), liquid samples (sea cucumber wine), defatted feces, and undefatted feces. Supplemental Table S3 The coefficient of the regression model, which is about the correlation between OTUs with abundances ≥ 1% and the utilization rate of SCSP. Supplemental Table S4 Mass spectrometry analysis parameters. Supplemental Table S5 Feed components (per kg of feed). Supplemental Figure S1 MRM chromatogram of PMP-fucose in plasma samples from the Control group (A) and the SCSP group (B), in urine samples from the Control group (C) and the SCSP group (D), and in fecal samples from the Control group (E) and the SCSP group (F). Supplemental Figure S2 The standard curve of PMP-fucose. Supplemental Figure S3 OPLS-DA of metabolites in feces detected in the positive (A) and negative (B) modes. Supplemental Figure S4 The metabolism pathway analysis in serum (A) and urine (B) metabolites.
