# Supplementary material for: Interaction between a Sulfated Polysaccharide from Sea Cucumber and Gut Microbiota Influences the Fat Metabolism in Rats
Source: Foods. 2023 Dec 14;12(24):4476. doi: 10.3390/foods12244476 (PMC10743057; doi:10.3390/foods12244476)
Supplement: Supplementary file 1 [file foods-12-04476-s001.zip › foods-2747859-supplementary.pdf]

Supplementary Data

Interaction between a Sulfated Polysaccharide from Sea Cucumber and Gut Microbiota Influences the Fat Metabolism in Rats

Yujiao Zhang, Haoran Song, Zhengqi Liu, Chunqing Ai, Chunhong Yan, Xiuping Dong and Shuang Song *

Liaoning Key Laboratory of Food Nutrition and Health,
Collaborative Innovation Center of Seafood Deep Processing,
National Engineering Research Center of Seafood, School of Food Science and Technology, Dalian Polytechnic University, Dalian 116034, China
* Correspondence: songshuang@dlpu.edu.cn

Supplemental Results

1 The validation of the method for quantification of SCSP

Supplemental Tables

Supplemental Table S1 The LOD and LOQ of solid samples (dried sea cucumber) and liquid samples (sea cucumber wine).

Supplemental Table S2 The recoveries of SCSP in solid samples (dried sea cucumber), liquid samples (sea cucumber wine), defatted feces, and undefatted feces.

Supplemental Table S3 The coefficient of the regression model, which is about the correlation between OTUs with abundances ≥ 1% and the utilization rate of SCSP.

Supplemental Table S4 Mass spectrometry analysis parameters.

Supplemental Table S5 Feed components (per kg of feed).

Supplemental Figures

Supplemental Figure S1 MRM chromatogram of PMP-fucose in plasma samples from the Control group (A) and the SCSP group (B), in urine samples from the Control group (C) and the SCSP group (D), and in fecal samples from the Control group (E) and the SCSP group (F).

Supplemental Figure S2 The standard curve of PMP-fucose.

Supplemental Figure S3 OPLS-DA of metabolites in feces detected in the positive (A) and negative (B) modes.

Supplemental Figure S4 The metabolism pathway analysis in serum (A) and urine (B) metabolites.

Supplemental Results

S1 The Validation of the Method for Quantification of SCSP

S1.1 Introduction of the Method

Fucose (Fuc) is the major monosaccharide component in SCSP. In the present study, SCSP was quantified by detecting Fuc released from SCSP through acid hydrolysis after PMP derivation. The typical MRM chromatograms of PMP-labelled Fuc are shown in Supplemental Fig. 1, and the mass spectrometry analysis parameters are shown in Supplemental Table 4.

S1.2 Linearity

The HPLC-MS/MS method showed excellent linearity ($y=10^7x-43475$) over the entire concentration range (0.005 mg/mL–0.4 mg/mL) for Fuc, with the correlation coefficient (R) being more than 0.99 (Supplemental Fig. 2).

1.3 Limit of Detection (LOD) and Limit of Quantification (LOQ)

Limit of detection (LOD) and limit of quantification (LOQ) were obtained on the basis of the signal to noise (S/N) ratio of 3 and 10, respectively. As shown in Supplemental Table 1, the LOQs of solid samples (dried sea cucumber) and liquid samples (sea cucumber wine) were 227 mg/kg and 0.014 mg/L for Fuc, respectively, indicating satisfactory sensitivity of the method under the present MS condition.

S1.4 Recovery

To determine the accuracy of the proposed method, the analytical recoveries were determined after spiking of a known amount of SCSP in solid samples (dried sea cucumber), liquid samples (sea cucumber wine), defatted feces, and undefatted feces. As shown in Supplemental Table 2, recoveries of 99% ~ 103.3% were observed. This indicates the quantitative method has good accuracy in various biological samples.

S1.5 Repeatability

The precision of the method was evaluated by repeatability study. The repeatability was expressed as the relative standard deviation (RSD) of the six identical samples. The RSD values of solid samples (dried sea cucumber) and liquid samples (sea cucumber wine) were 6.7% and 10.0% for Fuc, respectively, indicating acceptable repeatabilities.

Supplemental Tables

Supplemental Table S1 The LOD and LOQ of solid samples (dried sea cucumber) and liquid samples (sea cucumber wine).

Samples	Saccharides	LOD	LOQ
solid samples	Fuc	61.8mg/kg	227 mg/kg
liquid samples	Fuc	0.0042mg/L	0.014 mg/L

Supplemental Table S2 The recoveries of SCSP in solid samples (dried sea cucumber), liquid samples (sea cucumber wine), defatted feces, and undefatted feces.

Samples	Recovery (%)	Spiked amount
solid samples	99.0	69000 mg/kg
liquid samples	103.3	5 mg/L
defatted feces	103.0	8350mg/kg
undefatted feces	102.0	8350mg/kg

Supplemental Table S3 The coefficient of the regression model, which is about the correlation between OTUs with abundances ≥ 1% and the utilization rate of SCSP.

Model		Nonstandard coefficient		Standard regression coefficient	T	Significance
		B	Standard error	Beta		
1	(Constant)	-0.145	0.146		-0.991	0.360
	OTU_15	35.164	9.567	0.737	3.676	0.010
	OTU_57	38.257	14.928	0.514	2.263	0.043

Supplemental Table S4 Mass spectrometry analysis parameters.

Target compounds	Molecular ion (m/z)	Product ions (m/z)	Impact time (msec)	Collision energy (volts)	Declustering potential (volts)
2PMP-Fuc	495.2	373.2	150	40	80
		175.0*	150	70	100
2PMP-Lac	673.3	511.2	150	70	100
		271.0*	150	70	100

Supplemental Table S5 Feed components (per kg of feed)

Component	Content (/kg)
Crude proteins	≥180 g
Crude fats	≥40 g
Crude fiber	≤50 g
Crude ash	≤80 g
Moisture	≤100 g
Ca	10~18 g
P	6~12 g
Ca:P	1.2:1
Vitamin B1	≥8 mg
Vitamin B2	≥10 mg
Vitamin B6	≥6 mg
Lysine	≥8.2 g
Methionine+ Cystine	≥5.3 g
Arginine	≥9.9 g
Histidine	≥4 g
Fe	≥100 mg
Mn	≥75 mg
Cu	≥10 mg
Zn	≥30 mg
Vitamin A	≥7000 IU

Vitamin D	≥800 IU
Vitamin E	≥60 IU
Vitamin K	≥3 mg
Vitamin B12	≥0.02 mg

Supplemental Figures

Supplemental Figure S1

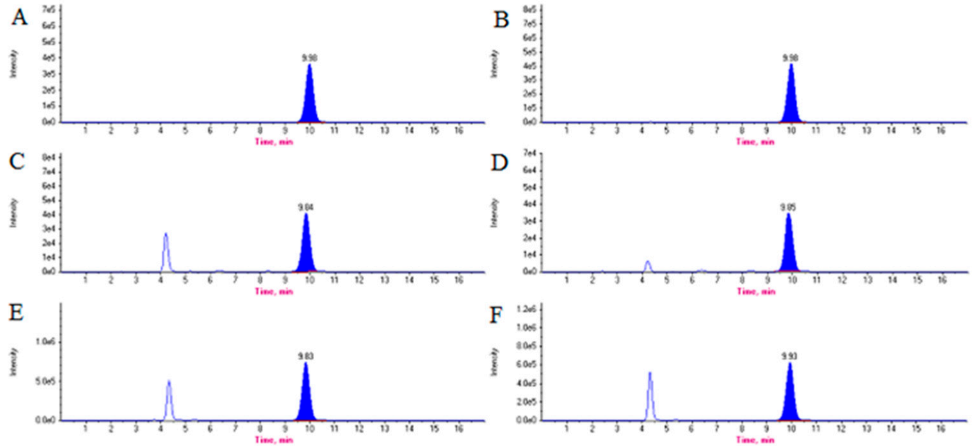

Supplemental Figure S1 MRM chromatogram of PMP-fucose in plasma samples from the Control group (A) and the SCSP group (B), in urine samples from the Control group (C) and the SCSP group (D), and in fecal samples from the Control group (E) and the SCSP group (F).

Supplemental Figure S2

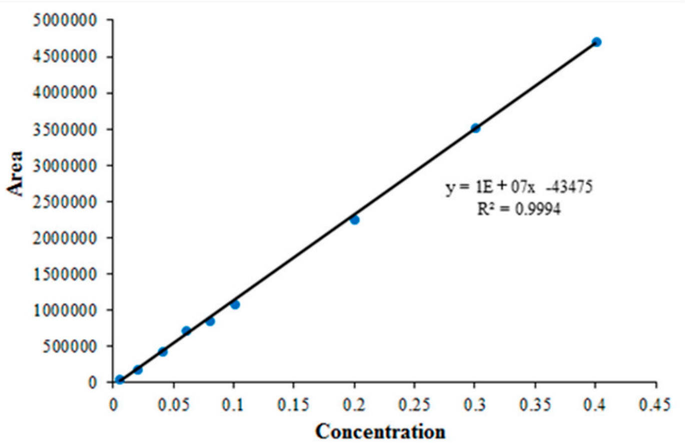

Supplemental Figure S2 The standard curve of PMP-fucose.

Supplemental Figure S3

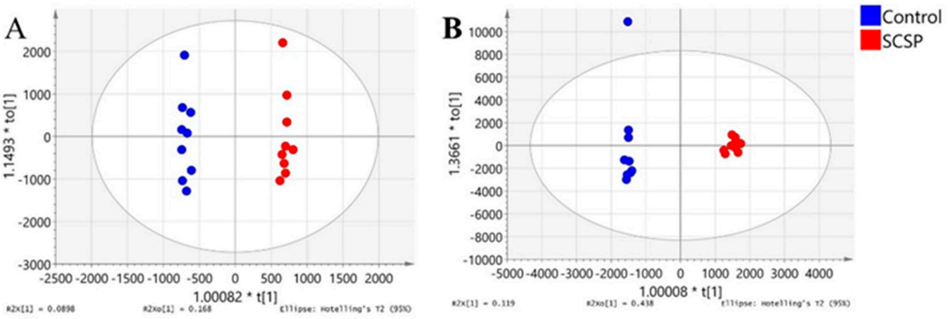

Supplemental Figure S3 OPLS-DA of metabolites in feces detected in the positive (A) and negative (B) modes.

Supplemental Figure S4

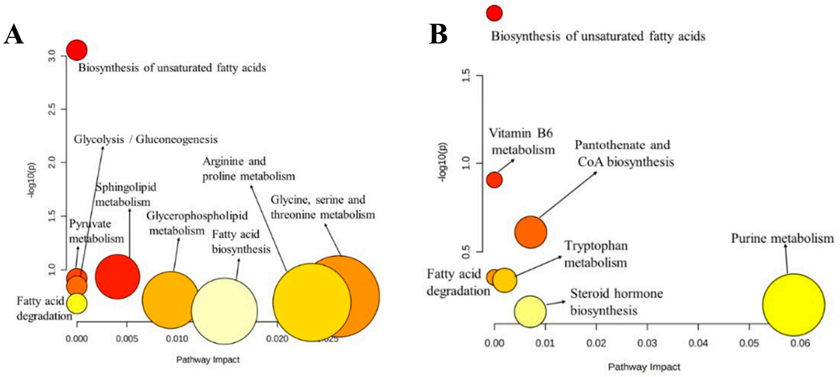

Supplemental Figure S4 The metabolism pathway analysis in serum (A) and urine (B) metabolites.
